# Autologous tenocyte stem cell injection for chronic tendinosis secondary to ruptured Achilles tendon repair: a case study

**DOI:** 10.1186/1757-1146-8-S2-O27

**Published:** 2015-09-22

**Authors:** Peter Manuel

**Affiliations:** 1 Australasian College of Podiatric Surgeons, East Melbourne, VIC 3002, Australia

**Keywords:** Tendinopathy, Achilles, tenocyte, surgery

## Background

Achilles tendinopathy is a common pathology with limited treatment options. This case study displays a novel approach using autologous tendon stem cells (tenocytes) injected into a chronically affected Achilles tendon following a failed repair of an Achilles tendon rupture.

## Process

Tenocytes were harvested from the patient's patella tendon and prepared using Orthocell regeneration technology. The tenocytes were multiplied and placed into an injectable scaffold, then injected under ultrasound guidance into the diseased tendon.

## Findings

The patient ruptured the Achilles tendon working as a nurse and underwent an orthopaedic surgery primary repair. At 6 months post operatively the patient had developed chronic tendinopathy affecting over 80% of the Achilles tendon. The patient was referred for opinion and had been off work for over 6 months with limitation in activities of daily living.

The patient underwent a surgical excision of a posterior calcaneal exostosis and debridement of the diseased Achilles tendon and harvest of tenocytes. 13 weeks post-surgery the tenocytes were injected into the Achilles tendon. The patient returned to full time work and normal activities of daily living at 8 weeks post injection. MRI studies were performed pre and post injection to assess diseased state of the tendon.

## Conclusions

Autologous tenocyte injection provides the clinician with a tool for treatment of chronic tendinopathy, where other treatment options have failed.

## Conflict of interest

Mr Peter Manuel has no financial relationship with Orthocell nor received any funding for this case study.

